# Sphingosine 1-phosphate receptor 1 modulators exert neuroprotective effects in central nervous system disorders

**DOI:** 10.3389/fphar.2025.1516991

**Published:** 2025-05-14

**Authors:** Shouming Chen, Lan Wu, Bingchen Lang, Guoyan Zhao, Wensheng Zhang

**Affiliations:** ^1^ Department of Anesthesiology, West China Second University Hospital, Sichuan University, Chengdu, Sichuan, China; ^2^ Key Laboratory of Birth Defects and Related Diseases of Women and Children (Sichuan University), Ministry of Education, Chengdu, China; ^3^ Department of Pharmacy, West China Second University Hospital, Sichuan University, Chengdu, Sichuan, China; ^4^ Department of Anesthesiology, West China Hospital, Sichuan university, Chengdu, Sichuan, China; ^5^ Laboratory of Anesthesia and Critical Care Medicine, National-Local Joint Engineering Research Centre of Translational Medicine of Anesthesiology, West China Hospital, Sichuan University, Chengdu, China

**Keywords:** S1P, S1PR, CNS, MS, NDDs, brain injury

## Abstract

The sphingosine 1-phosphate (S1P) signaling pathway has important and diverse functions. S1P receptors (S1PRs) are involved in the regulation of lymphocyte trafficking, cardio-cerebral function, vascular permeability, and bronchiolar tone, and have been recognized as therapeutic targets for a variety of diseases. A number of drugs related to the S1P signaling pathway have been approved for clinical use in the treatment of multiple sclerosis, and many similar drugs are also currently being tested in clinical trials at various stages. It appears that S1PR modulators may not only be useful for the treatment of multiple sclerosis, but may also have therapeutic effects on other types of central nervous system (CNS) disorders. The present review focuses on the therapeutic potential of S1PR1 modulators for treating disorders of the CNS.

## 1 Introduction

Lipids are mainly involved in energy storage and composition of cell membranes. The diversity of membrane lipid structures ensures that they are flexible and robust enough to adapt to different environments (Cartier and Hla, 2019). Among the membrane lipids, sphingolipids, including sphingomyelin and its metabolites, are an integral part of all cell membrane and myelin structures in the nervous system (Chatzikonstantinou et al., 2021; Coelho et al., 2010). Sphingosine 1-phosphate (S1P) is a pleiotropic sphingolipid produced via metabolism of sphingolipids (Chen et al., 2022). It was discovered by Sarah Spiegel’s team in the early 1990s and described as a potent secondary messenger with a function similar to that of diacylglycerol and Ca^2+^ (Cartier and Hla, 2019; Chen et al., 2022). However, it was not until 1997 that specific, high-affinity G protein-coupled receptors for S1P were detected, after which their multiple physiological roles in the human body gradually gained widespread attention (Chatzikonstantinou et al., 2021). There are five subtypes of S1P receptors (S1PRs): S1PR1, S1PR2, S1PR3, S1PR4, and S1PR5 (Chen et al., 2022). In many tissues, intracellularly generated S1P is rapidly degraded by the endoplasmic reticulum–resident S1P lyase, resulting in very low intracellular S1P concentrations (Cartier and Hla, 2019). In contrast, some S1Ps are transported out of the cell by binding to specific transporters to play extracellular roles (Pérez-Jeldres et al., 2021). In the extracellular milieu, S1Ps can bind to S1PRs on the cell membrane in an autocrine or paracrine manner to exert physiological or pathological effects (Cartier and Hla, 2019). For example, S1Ps help regulate risk factors for diseases related to the central nervous system (CNS), cardiovascular system, lung, liver, and cancer (Cartier and Hla, 2019; Chun et al., 2021). In addition, S1PRs have been reported to be involved in the regulation of lymphocyte trafficking and immune function (Hla, 2004; Jenne et al., 2009), heart rate (Sanna et al., 2004), vascular and bronchial tone (Hla et al., 2008; Roviezzo et al., 2007), membrane barrier permeability (Xiang et al., 2021; Stepanovska et al., 2020), microglial activation, neural axon growth, neuronal plasticity and synapse formation (Rosen et al., 2009), and atherosclerosis (Dumitrescu et al., 2023; Okajima, 2002).

S1PR1 was the first S1PR to be reported by Lee in 1998 (Lee et al., 1998). S1PR1 is expressed in many tissues, especially within the cardiovascular and immune systems (Chun et al., 2010; O'Sullivan and Dev, 2013). S1PR1 is also expressed in a variety of cells in the CNS, where it regulates various cellular functions, such as the growth of nerve axons, neuronal plasticity, synapse formation, cell migration, neurotransmission, and apoptosis (Groves et al., 2013; Nitzsche et al., 2021). Furthermore, S1PR1 is involved in the neuroinflammatory response in a variety of diseases, such as Alzheimer’s disease (Zhu et al., 2023), experimental autoimmune encephalomyelitis (Tsai et al., 2016; Zheng et al., 2023; Uchi et al., 2023; Garris et al., 2013), cancer-induced neuroinflammation (Grenald et al., 2017), and COVID-19 – induced neurological dysfunction (Pan et al., 2021).

**FIGURE 1:**
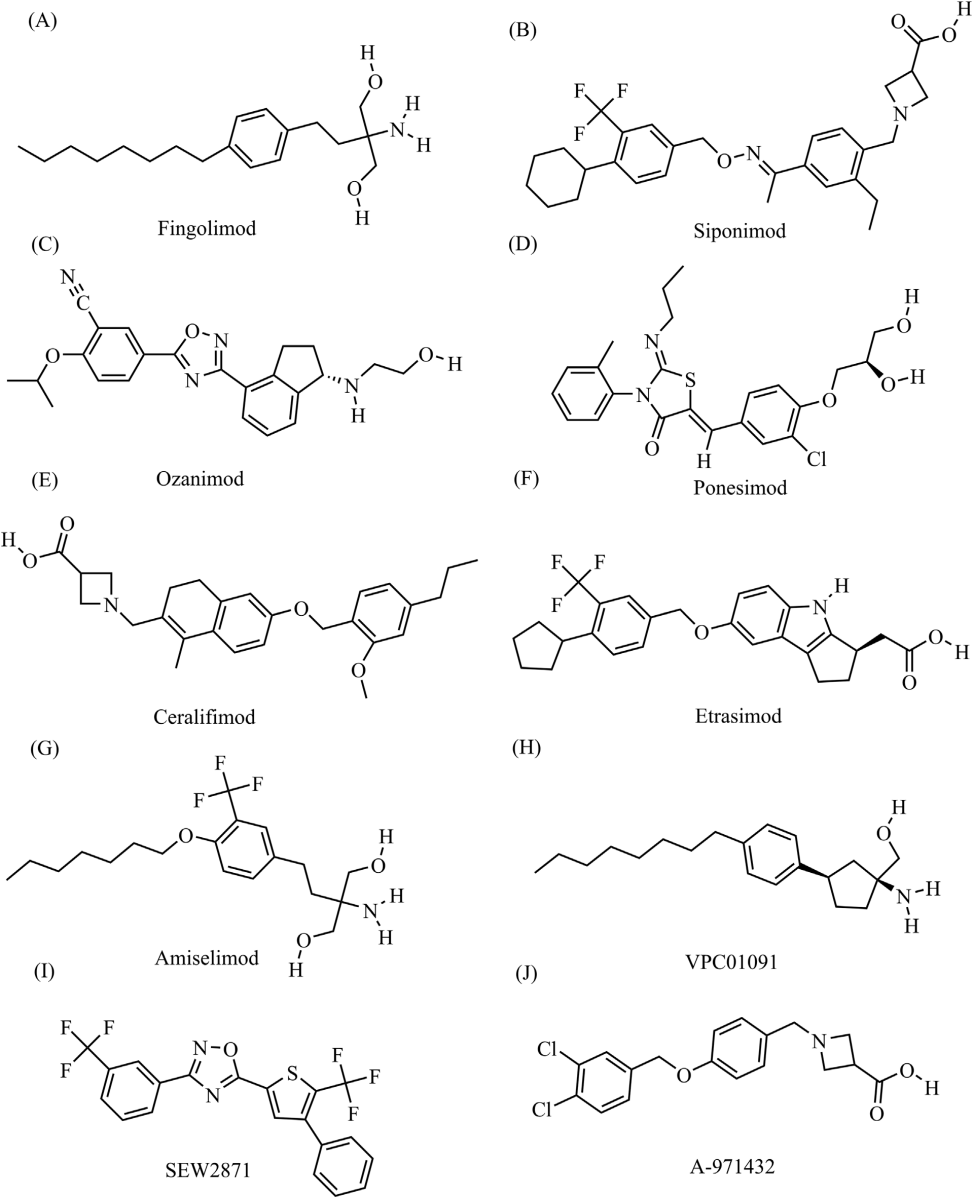
The chemical structures of S1PR1 modulators **(A)** Fingolimod, C_19_H_33_NO_2_, molecular weight, 307.5 g/mol **(B)** Siponimod, C_29_H_35_F_3_N_2_O_3_, molecular weight, 516.6 g/mol; **(C)** Ozanimod, C_23_H_24_N_4_O_3_, molecular weight, 404.5 g/mol **(D)** Ponesimod, C_23_H_25_ClN_2_O_4_S, molecular weight, 461.0 g/mol; **(E)** Ceralifimod, C_27_H_33_NO_4_, molecular weight, 435.6 g/mol **(F)** Etrasimod, C_26_H_26_F_3_NO_3_, molecular weight, 457.5 g/mol; **(G)** Amiselimod, C_19_H_30_F_3_NO_3_, molecular weight, 377.4 g/mol **(H)** VPC01091, C_20_H_33_NO, molecular weight, 303.5 g/mol; **(I)** SEW2871, C_20_H_10_F_6_N_2_OS, molecular weight, 440.4 g/mol **(J)** A-971432, C_18_H_17_Cl_2_NO_3_, molecular weight, 366.2 g/mol. All chemical structures and related data in this section were obtained from the PubChem database (https://pubchem.ncbi.nlm.nih.gov/).

Clinically available S1PR modulators are a novel class of immunosuppressive agents that act as S1PR functional antagonists or agonists (McGinley and Cohen, 2021). Competitive antagonists of S1PRs (Maruyama et al., 2024; Fujii et al., 2012) exert their effects by occupying the binding pocket, which distinguishes their mechanism of action from that of agonists. The first S1PR1 modulator to be developed was fingolimod (FTY720), which was synthesized by the research group of Tetsuro Fujita at Kyoto University in 1992 while investigating structure activity relationships of derivatives of the fungal metabolite myriocin (ISP-I), isolated from *Isaria sinclairii* (Strader et al., 2011). Fingolimod, an analog of sphingosine, non-selectively binds to S1PRs, and potent inhibitor of sphingolipid synthesis that is currently approved for the treatment of multiple sclerosis (MS) and has both antifungal and immunosuppressive properties (McGinley and Cohen, 2021; McEvoy et al., 2020; Tsai and Han, 2016). Fingolimod is also being tested in basic and clinical studies for its potential utility in treating hemorrhagic and ischemic stroke (; Zhao et al., 2024; Kraft et al., 2013), amyotrophic lateral sclerosis (Potenza et al., 2016; Berry et al., 2017), and chronic inflammatory demyelinating polyneuropathy (Hughes et al., 2018). Fingolimod can bind to and exert effects through S1PR1, S1PR3, S1PR4, and S1PR5. However, receptor subtype selectivity for S1PR1 is theoretically favored to minimize safety concerns related to interaction with other S1PR subtypes. This specificity helps mitigate the primary safety issue related to cardiac side effects, including bradycardia, which necessitates extended monitoring after the initial dose. As a result, it has enabled the development of smaller compounds with shorter half-lives, faster onset of action without the need for phosphorylation to activate, and maintained therapeutic effectiveness (Hughes et al., 2018). Therefore, second-generation S1PR1 modulators were developed, including siponimod (BAF312), ozanimod (RPC1063), and ponesimod (ACT-128800) (Chun et al., 2021; Dumitrescu et al., 2023; McGinley and Cohen, 2021). These drugs are more selective derivatives of fingolimod that theoretically have less effect on other S1PR subtypes, thereby improving their safety profiles (Roy et al., 2021). Ozanimod has been approved for the treatment of MS and was recently also approved for the treatment of moderate-to-severe ulcerative colitis (Filippi et al., 2018; Lamb, 2020). In addition, ongoing clinical trials are testing the use of ozanimod for the treatment of Crohn’s disease, systemic lupus erythematosus, and COVID-19 (Lellouche et al., 2024; Taylor Meadows et al., 2018; Sleutjes et al., 2020). Many other S1PR1 modulators are still in the clinical trial phase, including ceralifimod (ONO-4641), cerenimod (ACT-334441), etrasimod (APD334), amiselimod (MT-1303), VPC01091, and VPC23019a (Figure 1). (Chun et al., 2021; McGinley and Cohen, 2021) While ceralifimod and amiselimod showed positive results in phase II trials, they are no longer being studied further for their use in MS due to strategic market realignment of the companies that developed them (Roy et al., 2021).

The blood-brain barrier (BBB), which consists of endothelial cells, pericytes, astrocytes, and microglia, protects the CNS from harmful circulating substances (Benz and Liebner, 2022). Dysregulation of S1P signaling disrupts the BBB in the early stages of many CNS disorders, including MS, Alzheimer’s disease (AD), and stroke (McGinley and Cohen, 2021). This review focuses on the current state of treatment of neurological diseases with S1PR1 modulators (Tables 1, 2).

**TABLE 1:** The application of S1PR1 modulators in animal models of CNS diseases.

Modulator name	Condition treated	Animal model	Dosage	Time taken	Effects
Fingolimod (Choi et al., 2011)	MS, Experimental autoimmune encephalo myelitis (EAE)	Mice	i.p., 3 mg/kg	A week	Fingolimod has been shown to alleviate symptoms in the EAE model, potentially through its action on the S1PR1 receptor located on astrocytes rather than on neurons
Fingolimod (Smith et al., 2018)	MS, EAE	Female Mice	Oral gavage 1 mg/kg	Daily, until termination of the experiment	Significantly ameliorated brain tissue atrophy in the cerebellum and striatum
Ponesimod (Zhu et al., 2023)	AD, 5XFAD mice	Male and female Mice	Oral gavage 30 mg/kg	Once a day for 4 weeks	Reduced the levels of TNF-α and CXCL10; increased the level of IL-33; and improved spatial memory
Fingolimod (Rolland et al., 2013)	ICH	Male mice	i.p., 1 mg/kg	1 h after ICH-induction or daily administrations (1h, 24h, and 48 h after ICH-induction)	Mice: decreased expression of ICAM1, interferon γ, and interleukin-17 Rat: reduced spatial and motor learning deficits, brain atrophy and neuronal cell loss at 8–10 weeks after ICH.
		Male rat			
Fingolimod (Rolland et al., 2017)	GMH	Male and female rats	i.p., 0.25 or 1.0 mg/kg	1, 24, and 48 h after GMH	Activates the S1PR/Akt/Rac1 signaling pathway; alleviates cerebral edema, reduces blood-brain barrier permeability, and improves long-term neurological damage and behavioral deficits in rats
SEW2871 (Rolland et al., 2017)	GMH	Male and female rats	i.p., 5 mg/kg	1, 24, and 48 h after GMH	Significantly enhanced long-term neurocognitive function, reduced brain tissue loss, and decreased brain water content
Siponimod (Zhang et al., 2023)	ICH	Mice	i.p., 1 mg/kg	30 min, 24 h and 48 h after ICH	Reduced brain tissue volume loss, edema, and long-term atrophy; inhibited neuronal degeneration; and improved neurological function
Siponimod (Bobinger et al., 2019)	ICH	Mice	i.p., 0.3 or 3 mg/kg	30 min after ICH or 30 min, 24h and 48 h after ICH	Reduced perihemorrhagic edema, improved neurological deficits, and minimized weight loss
SEW2871 (Iwasawa et al., 2018)	tMACO	Mice	i.p., 5 or 1.5 mg/kg	3 or 7 days	Increased the number of leptomeningeal collateral arteries, enhanced cerebral blood flow, reduced infarct volume, and improved neurologic function
SEW2871 (Ichijo et al., 2015)	Unilateral common carotid occlusion (CCAO) and permanent MACO	Mice	i.p., 5 mg/kg	7 days	CCAO: S1PR1 expression colocalized with endothelial cell markers in the leptomeningeal arteries and significantly increased on the side of the CCAO. Additionally, S1PR1 selective agonists markedly enhanced cerebral blood flow (CBF) and dilated the diameter of leptomeningeal collateral vessels. pMACO: Reduced infarct volume and improved functional recovery.
LASW1238 (Brait et al., 2016)	tMACO	Male mice	i.p., 3 or 10 mg/kg	Once	10 mg/kg demonstrated a significantly smaller infarct volume compared to both 3 mg/kg and the vehicle group
Siponimod (Huang et al., 2022)	tMACO	Mice	i.p., 0.6 mg/kg	From day 3 after MACO to end of the experiment	Siponimod improves recovery of neurological function
Fingolimod (Hasegawa et al., 2010)	tMACO	Rat	i.p., 0.25 or 1 mg/kg	Once	Significantly reduced infarct volume and improved neurological scores at 24 and 72 h post-MCAO compared to the vehicle group
SEW2871 (Hasegawa et al., 2010)	tMACO	Rat	i.p., 5 mg/kg	Once	Significantly reduced infarct volume and improved neurological score at 24 and 72 h after MCAO compared with the vehicle group
Fingolimod (Doi et al., 2013)	AD	Mouse, Primary cortical neurons	1–100 pM	3 h	Enhanced the expression of brain-derived neurotrophic factor (BDNF) in neurons
Fingolimod (Motyl et al., 2018)	PD	Male mice	i.p., 1 mg/kg	Daily for 10 consecutive days	Fingolimod enhanced locomotor performance by activating the pro-survival enzyme Akt kinase and promoting the phosphorylation of BAD proteins, thereby potentially protecting mitochondria through the reduction of pro-apoptotic signaling
Fingolimod (Zhao et al., 2017)	6-OHDA induced PD	Male mice	i.p., 0.5 or 1 mg/kg	Daily for 21 consecutive days	Fingolimod significantly alleviated motor function deficits in both PD mouse models, reduced the loss of tyrosine hydroxylase-positive neurons in the substantia nigra, decreased striatal dopamine and its metabolite levels, maintained ERK phosphorylation levels, and reduced cleaved caspase 3 expression
	Rotenone induced PD			Daily for 28 consecutive days	
Fingolimod and SEW2871 (Pépin et al., 2020)	PD	Male mice	Oral, fingolimod 1 mg/kg; SEW2871 20 mg/kg	Daily for 14 consecutive days	Protects against the loss of dopaminergic neurons and motor deficits, and demonstrates the ability to prevent neuroinflammation, including the activation of astrocytes and microglia, as well as the reduction of BDNF levels in key brain regions associated with motor functions
Fingolimod (Miguez et al., 2015)	HD	Male mice	i.p., 0.3 mg/kg	Every 4 days for 12 weeks	Ameliorated long-term memory deficits and dendritic spine loss in CA1 hippocampal neurons, prevented astrogliosis and overactivation of nuclear factor kappa beta (NF-κB) signaling in the R6/1 hippocampus, reduced tumor necrosis factor alpha (TNFα), and increased cAMP levels while promoting the phosphorylation of CREB and RhoA in the hippocampus
Fingolimod (Potenza et al., 2016)	ALS	Female and male mice	i.p., 0.1 or 1 mg/kg	Three times a week until the end stage of the disease	The drug improved the neurological phenotype and extended survival, which was associated with significant modulation of neuroinflammatory and protective genes (CD11b, Foxp3, iNOS, IL 1β, IL 10, Arg1, and Bdnf) in the motor cortex and spinal cord of the animals
Siponimod (Cuzzocrea et al., 2018)	TBI	Male mice	i.p., 1 mg/kg	1 h and 4 h after trauma	Exhibited anti-inflammatory and immunomodulatory effects in TBI mice, inhibited astrocyte and microglial activation, reduced cytokine release, reversed the decline in adhesion factor expression, and suppressed T-cell activation by decreasing CD4^+^ and CD8^+^ expression, thereby reducing the area of brain damage and preserving the normal structure of brain tissue
CYM-5442 (Zhang et al., 2022)	TBI	Mice	i.p., 3 mg/kg	Daily	Significantly reduced brain edema and neurological deficits, while also transiently inhibiting lymphocyte migration without causing a sustained reduction in lymphocyte levels
Fingolimod (Mencl et al., 2014)	TBI	Mice	Intravenous injection 1 mg/kg	Once	Significantly reduced the number of circulating lymphocytes and attenuated immune cell infiltration into the damaged brain parenchyma, but did not reduce the extent of brain injury or improve neurological deficits

**TABLE 2:** The application of S1PR1 modulators in clinical practice and clinical research for the treatment of CNS diseases.

Modulator name	Country	Condition treated	Number of patients	Time taken	Effects
Fingolimod (Dumitrescu et al., 2023)	United States of America European Union	MS	None	Adults: one 0.5 mg capsule, orally, once daily Pediatric with body weight ≤40 kg: one 0.25 mg capsule, orally, once daily Pediatric >40 kg: one 0.5 mg capsule, orally, once daily	
Siponimod (Dumitrescu et al., 2023)	United States of America European Union	MS	None	Adults: one 2 mg tables, orally, once daily. genotypes CYP2C9*2*3 or 1*3: 1 mg daily. Titration: over 5 days, from 0.25 mg qd to 1.25 mg qd	
Ozanimod (Dumitrescu et al., 2023)	United States of America European Union	MS	None	Adults: one 0.92 mg capsule, orally, once daily Titration: over the course of 7 days, from 0.23 mg qd to 0.46 mg qd	
Ponesimod (Dumitrescu et al., 2023)	United States of America European Union	MS	None	Adults: one 20 mg tablet, orally, once daily Titration: over the course of 2 weeks, from 2 to 10 mg qd	
Fingolimod (Golan et al., 2019)	Israel	MS	21	None	Enhanced BDNF secretion from T cells
Fingolimod (Fu et al., 2014)	China	Acute ischemic stroke (AIS)	22	0.5 mg/day orally for 3 days	Fingolimod recipients had lower circulating lymphocyte counts, milder neurological deficits, and better recovery of neurological functions
Fingolimod (Liantao et al., 2019)	China	AIS	90	0.5 mg/day orally for 3 days	At 90 days post-treatment, the National Institutes of Health Stroke Scale (NIHSS) score and modified Rankin scale (mRS) score in the fingolimod group were significantly lower than those in the control group, while the Barthel index (BI) was significantly higher
Fingolimod (Tian et al., 2018)	China	AIS	46	0.5 mg/day orally for 3 days	Fingolimod combined with alteplase improved early clinical outcomes at 24 h and mRS distribution at 90 days, reduced perfusion lesions, suppressed infarct growth, enhanced anterograde reperfusion, and prevented retrograde reperfusion failure
Fingolimod (Zhu et al., 2015)	China	AIS	47	0.5 mg/day orally for 3 days	Reduced circulating lymphocytes, smaller lesion volumes, less hemorrhage, and attenuated neurological deficits as measured by the NIHSS score. No serious adverse events were observed in any patients
Fingolimod (Berry et al., 2017)	United States of America	ALS	28	0.5 mg/day orally for 4 weeks	No serious adverse events were observed. Circulating lymphocytes decreased significantly in the fingolimod group. Additionally, nine immune-related genes, including forkhead box P3 and CD40 ligand, were significantly downregulated in the fingolimod group

## 2 Multiple sclerosis

MS is an immune-mediated disease of the CNS, in which inflammatory demyelination and axonal loss produce a range of neurologic symptoms (Klotz et al., 2023). The etiology of MS is unclear and may be related to a variety of factors, including genetics, the environment, and viral infections (Ghasemi et al., 2017). MS typically occurs in young adults and is more common in females, with a male-to-female ratio of 1:3 in most developed countries (Orton et al., 2006). Various parts of the CNS can be affected, and the clinical manifestations are diverse. Common symptoms include vision loss, diplopia, limb sensory disorder, limb motor disorder, ataxia, and bladder or rectal dysfunction. The disease is categorized into relapsing-remitting MS, secondary-progressive MS, primary-progressive MS, and other types (Lublin et al., 2014).

Currently, approved S1PR1 receptor modulators for the treatment of MS include fingolimod, siponimod, ozanimod, and ponesimod (McGinley and Cohen, 2021; Lamb, 2020; Markham, 2021; Alnaif et al., 2023). Fingolimod has been approved for marketing many countries and regions, including the United States, the European Union and the United Kingdom, China, Japan, and India, making it one of the important drugs for the treatment of MS. Fingolimod is a prodrug that requires phosphorylation by intracellular sphingosine kinases to gain affinity for S1PRs (Brinkmann et al., 2002). Phosphorylated fingolimod acts as a functional antagonist of S1PR1 expressed on lymphocytes, sequestering these cells in lymph nodes and preventing them from infiltrating the CNS, which is one of the mechanisms of action by which it ameliorates the symptoms of MS (Brinkmann et al., 2010; Ntranos et al., 2014). Choi et al. discovered that the therapeutic benefits of fingolimod on autoimmune encephalomyelitis (an animal model of MS) were absent in mice lacking S1PR1 expression in astrocytes, indicating that astrocytes play a crucial role in mediating the effects of fingolimod in MS (Choi et al., 2011). Additionally, when S1PR1 was knocked out in mice astrocytes, fingolimod lost its ability to prevent chemotherapy-induced cognitive impairment (Squillace et al., 2022). Singh et al. (2022) and Zhu et al. (2023) both found that S1PR1 modulators have a neuroprotective effect on mice glial cells. Golan et al. found that fingolimod treatment significantly increased the secretion of brain-derived neurotrophic factor (BDNF) by T cells in patients with MS after 6 and 12 months (Golan et al., 2019). Smith et al. found that fingolimod-mediated BDNF increases within the CNS may contribute to limiting progressive tissue loss during MS mice (Smith et al., 2018).

Siponimod, a second-generation S1PR modulator, is an alkoxyimine derivative of fingolimod. Its initial structure was optimized by means of chemical synthesis to increase its potency toward S1PR1 and selectivity for S1PR3, thereby increasing its therapeutic potential for MS and reducing the risk of side effects (Pan et al., 2013). The main mechanism by which siponimod treats MS is by reducing brain inflammation. Ozanimod is also a second-generation S1PR modulator that has been approved for the treatment of relapsing-progressive MS in the United States and the European Union (Dumitrescu et al., 2023). Ozanimod has a more complex *in vivo* metabolism process and a shorter half-life than siponimod (Chun et al., 2021; Rasche and Paul, 2018). Ponesimod is a highly selective S1PR1 antagonist with S1PR5 antagonistic activity and is currently approved for the treatment of MS in the United States, the European Union, and the United Kingdom (Rasche and Paul, 2018).

To investigate the most appropriate regimen for the treatment of MS using S1PR1 receptor modulators and to provide an evidence base for using this drug to treat patients with MS, investigators performed a reticulated meta-analysis of 13 randomized controlled studies (Tong et al., 2021). The results of the meta-analysis showed that fingolimod, siponimod, ozanimod, amiselimod, and ponesimod were all effective in reducing the annual MS recurrence rate compared to the placebo group. Amiselimod (0.4 mg) was the most effective treatment, while the treatment with the highest patient acceptance was ozanimod (1 mg) (Tong et al., 2021). Another study of a large number of clinical studies identified adverse effects of S1PR1 modulators, including decreased lymphocyte counts, increased hepatic aminotransferase concentrations, bradycardia and arrhythmias, macular edema, hypertension, recurrence of herpes zoster, and convulsions. No new complications other than these symptoms occurred with long-term (5 years) oral administration (Kappos et al., 2018).

## 3 Stroke

Stroke, which can be divided into ischemic stroke and hemorrhagic stroke (Campbell et al., 2019; Unnithan AKADas JMMehta, 2023), is a chronic non-communicable disease characterized by high morbidity, disability, mortality, recurrence, and heavy economic burden that is a leading cause of death and disability worldwide (Campbell and Khatri, 2020). Because of accelerated population aging and urbanization, the prevalence of stroke risk factors and the incidence of stroke are increasing (Yousufuddin and Young, 2019). Therefore, the prevention and treatment of stroke are of great significance to global health.

The inflammatory response following cerebral hemorrhage or cerebral ischemia leads to increased BBB permeability, cerebral edema, and neuronal cell death, resulting in neurological complications (Alsbrook et al., 2023). In mouse and rat animal model of cerebral hemorrhage, administration of fingolimod reduced lymphocyte infiltration into the parenchyma, decreased expression of ICAM1, interferon γ, and interleukin-17, reduced cerebral edema, and improved neurological prognosis (Rolland et al., 2013). Another group found that fingolimod activates the S1PR/Akt/Rac1 signaling pathway, which alleviates rat brain tissue edema, reduces blood-brain barrier permeability, and improves long-term brain damage and behavioral deficits following germinal matrix hemorrhage (GMH) in neonatal rats (Rolland et al., 2017). In a study using a mouse brain hemorrhage model, siponimod treatment significantly reduced brain tissue volume loss, edema, and long-term atrophy compared to controls, while also inhibiting neuronal degeneration and improving neurological function. These protective effects may result from downregulation of lymphocyte chemokines and helper T-cell expression, as well as reduced neutrophil and lymphocyte infiltration and attenuated T-cell activation in perihematomal tissues (Zhang et al., 2023). The protective effect of siponimod against hemorrhagic stroke was also demonstrated in a study by Bobinger et al. (2019), which showed that siponimod significantly reduced brain edema and the wet/dry brain tissue ratio in a mouse model of ICH. Furthermore, siponimod increased the survival rate of ICH mice and decreased their neurological deficits.

Current treatments for ischemic stroke focus on promoting reperfusion but are limited by reperfusion injury and the risk of hemorrhage (Zhang et al., 2024). Ideally, pharmacologic treatments for this condition would reduce brain damage and improve neurologic function. Researchers carried out middle cerebral artery embolization (MACO) in mice and found that (Lucaciu et al., 2020) S1P was expressed in specific significant concentration gradients in various organs after 24 h of MACO, with the lowest concentrations in the spleen, moderate concentrations in the circulation, and the highest concentrations in the ischemic core region, which the authors suggested may be associated with lymphocyte recruitment, while ceramide levels in the brain remained unchanged but S1PR expression was altered in MACO (Lucaciu et al., 2020). Therefore, the researchers concluded that differential S1PR expression in acute ischemic stroke may attract T lymphocytes toward the S1P gradient, which may also be the theoretical basis for the ability of S1PR modulators to treat ischemic stroke (Lucaciu et al., 2020). One study found that S1P expression is significantly upregulated in ischemic regions of the brain in the mouse MACO model, rising to a maximum around 14 days after occlusion (Kimura et al., 2008). Another study showed that S1PR1 expression on endothelial cells in the mice leptomeningeal arteries increased after MACO, peaking at 6 h, and that at 24 h S1PR1 expression in neurons began to increase significantly. Intraperitoneal administration of the S1PR1-selective agonist SEW2871 for 7 days after mice MACO resulted in an increased number of leptomeningeal collateral arteries, improved cerebral blood flow, reduced infarct volume, and improved neurologic function (Iwasawa et al., 2018). S1PR1 expression was also upregulated in leptomeningeal artery endothelial cells after unilateral common carotid occlusion in mice. Administration of SEW2871 to the mice resulted in increased cerebral blood flow (CBF), increased lateral cerebral vessel diameters, reduced infarct volume, and functional recovery superior to that seen in the control mice (Ichijo et al., 2015). LASW1238, another selective S1PR1 agonist, reduces infarct volume after ischemia/reperfusion in MACO model mice (Brait et al., 2016). In addition, siponimod improves recovery of neurological function in MACO model mice (Huang et al., 2022). Furthermore, fingolimod prevents apoptosis by activating Akt and ERK via S1PR1, thereby attenuating rat neuronal damage and improving neurobehavior after cerebral ischemia (Hasegawa et al., 2010).

In addition to animal studies, fingolimod has been assessed in clinical studies of subarachnoid hemorrhage and acute ischemic stroke (Fu et al., 2014; Liantao et al., 2019; Zhang et al., 2017; Zhu et al., 2015; Tian et al., 2018; Männer et al., 2020). A meta-analysis of five clinical randomized controlled studies found that fingolimod reduced the size of the cerebral infarct area and improved neurological recovery in patients with stroke (Bai et al., 2022). No significant difference in the incidence of complications and adverse events was found between the fingolimod group and the conventional treatment group. Furthermore, fingolimod did not cause immunodeficiencies in patients, owing to the generally shorter treatment period.

## 4 Neurodegenerative diseases

Neurodegenerative diseases (NDDs) are a group of disorders in which neurons in the central or peripheral nervous system fail to renew themselves efficiently, resulting in a progressive loss of neurons that eventually leads to impaired memory, cognitive, behavioral, sensory, and motor functions (Kovacs, 2017). They mainly include Alzheimer’s disease, Parkinson’s disease, Huntington’s disease, and amyotrophic lateral sclerosis. Recent studies have shown that S1P metabolism and signaling play important roles in the pathogenesis of NDDs, such as regulating cell survival, apoptosis, autophagy, and β-amyloid production and aggregation (Angelopoulou and Piperi, 2019).

### 4.1 Alzheimer’s disease

Alzheimer’s disease (AD) is the most common age-related neurodegenerative disease and often presents with dementia (Breijyeh and Karaman, 2020). The hallmark pathological changes that occur during AD are deposition of β-amyloid plaques and tau protein abnormalities (O'Sullivan and Dev, 2017). One study demonstrated a significant upregulation of S1P and S1PR1 expression in senescent 5xFAD mice (an AD model) compared to wild-type mice, along with enhanced activation of the Akt/mTOR/Tau signaling pathway, which is downstream of S1PR1 (Jung et al., 2023). Treating 5xFAD mice with fingolimod restored S1P and S1PR1 expression levels, as well as Akt/mTor/Tau signaling. This suggests that dysregulation of S1P and S1PR1 metabolism could be involved in AD development through regulation of the Akt/mTor/Tau signaling pathway (Jung et al., 2023). Joshi and Doi showed that fingolimod protects mice against β-amyloid–induced neuronal toxicity (Joshi et al., 2017; Doi et al., 2013), and that fingolimod may increase the expression of brain derived neurotrophic factor (BDNF), which then exerts neuroprotective effects through activation of the TrkB and ERK1/2 signaling pathways (Doi et al., 2013). Volkmar et al. and Efthalia et al. reviewed the theoretical basis and molecular mechanisms of whether fingolimod can be used to treat AD. (Angelopoulou and Piperi, 2019; Leßmann et al., 2023) Fingolimod can modulate many pathological mechanisms that are highly relevant to AD pathogenesis, for example, by inhibiting β-amyloid secretion and deposition, inhibiting apoptosis, and enhancing BDNF production. In addition, fingolimod can modulate neuroinflammation, protect against N-methyl-D-aspartate (NMDA)-mediated neuronal cell excitotoxicity, and modulate late glycosylation end-product signaling axis receptors (Angelopoulou and Piperi, 2019). Although the results of the current studies are promising, they are largely based on animal models. To investigate whether fingolimod has therapeutic potential for AD in humans, researchers used a network pharmacology study approach to explore possible fingolimod targets in the frontal cortex of patients with AD. (Yin et al., 2021) The results showed that fingolimod may regulate neuron and astrocyte numbers and function, GABA synaptic function, and miRNA interactions, decrease frontal cortical neutrophil infiltration and neuron apoptosis in patients with AD. (Yin et al., 2021) However, there are currently no clinical studies reported on the treatment of AD with fingolimod. Based on the results of current animal experiments, Volkmar et al. suggest that clinical research on fingolimod for AD treatment should be initiated as soon as possible, as expanding the indications for already marketed drugs is a faster, cheaper, and safer approach (Leßmann et al., 2023).

### 4.2 Parkinson’s disease

Parkinson’s disease (PD) is characterized by progressive loss of dopaminergic neurons, and effective treatments are limited (Zhou et al., 2023). In recent years, fingolimod has been increasingly reported to protect cells from damage by regulating S1PR1. To determine whether fingolimod protects against PD development, fingolimod was used to treat two different mouse models of PD. The results showed that fingolimod significantly attenuated motor function deficits in both PD mouse models, reduced the loss of nigral tyrosine hydroxylase-positive neurons, decreased striatal dopamine and metabolite levels, and maintained ERK phosphorylation levels while decreasing cleaved caspase 3 expression (Zhao et al., 2017). In a 1-methyl-4-phenyl-1,2,3,6-tetrahydropyridine (MPTP)-induced mouse model of PD, fingolimod improved locomotor performance by activating the pro-survival enzyme Akt kinase and promoted the phosphorylation of BAD proteins, which may protect mitochondria by reducing pro-apoptotic signaling (Motyl et al., 2018). Another study investigated the protective effects of different S1PR modulators on PD mice and showed that both the non-selective S1PR modulator fingolimod and the selective S1PR modulator SEW2871 protected against the loss of dopaminergic neurons, as well as the development of motor deficits, in a mouse model of PD. In addition, treatment with fingolimod and SEW2871 prevented astrocyte activation in the brains of PD mice and reversed the decrease in BDNF levels in brain regions involved in the control of motor function (Pépin et al., 2020).

### 4.3 Huntington’s disease

Huntington’s disease (HD) is an inherited neurodegenerative disorder characterized by motor and cognitive deficits that involves the striatum, cortex, and hippocampus (McColgan and Tabrizi, 2018). Fingolimod prevents memory loss and enhances synaptic plasticity in hippocampal neurons and can prevent BDNF receptor dysregulation in the mouse hippocampus by down-regulating TNF-α and the P75 neurotrophin receptor (p75NTR) (Miguez et al., 2015). Another study reported that treatment with A-971432 (0.1 mg/kg) maintained normal body weight, prevented the development of progressive motor deficits, delayed the onset of disease symptoms, and significantly prolonged survival time in HD model mice. The exact mechanism is unclear, but may be related to the ability of A-971432 to activate/phosphorylate the pro-survival kinases AKT and ERK in the brain (Di Pardo et al., 2018).

### 4.4 Amyotrophic lateral sclerosis

Amyotrophic lateral sclerosis (ALS) is a multifactorial disease characterized by progressive degeneration of motor neurons in the spinal cord and motor cortex (Mead et al., 2023). Riluzole was the first drug approved for the treatment of ALS, and although many new ALS drugs are already in phase II and phase III clinical trials, they all appear to have limited therapeutic efficacy at this time (Xu et al., 2021). Animal experiments and clinical trials have been conducted to assess the efficacy and safety of fingolimod for the treatment of ALS. A study using mSOD1G93A ALS model mice demonstrated that administration of fingolimod attenuated neurologic deficits, prolonged survival time, improved motor performance, and upregulated Arg1, IL-10, and BDNF expression compared to control mice (Potenza et al., 2016). The results from a clinical phase IIa trial, a randomized controlled study designed to test the short-term safety, tolerability, and therapeutic targets of fingolimod in patients with ALS, showed that fingolimod is safe and well tolerated and can reduce the number of circulating lymphocytes (Berry et al., 2017).

## 5 Traumatic brain injury

Traumatic brain injury (TBI) is the leading cause of death and disability in developed countries, with approximately 10 million people worldwide experiencing TBI each year (Zou et al., 2021). In 2013, the annual mortality rate for TBI in China was 12.99/100,000, implying that approximately 18,000 people die from TBI each year in the country (Cheng et al., 2017), and the death rate is still rising. In recent years, studies have shown that TBI induces neuroinflammation and immune cell infiltration into the brain (El Baassiri et al., 2024). This provides a theoretical basis for the treatment of TBI with S1PR1 modulators. One study suggested that siponimod and TASP0277308 (a S1PR1 competitive antagonist, 1 mg/kg) have anti-inflammatory and immunomodulatory effects in TBI mice; in particular, they inhibit astrocyte and microglial cell activation, reduce cytokine release, reverse the decline in adhesion factor expression, and reduce T-cell activation by decreasing CD4^+^ and CD8^+^ expression, thereby reducing the area of brain damage and maintaining the normal structure of brain tissue and neuronal plasticity (Cuzzocrea et al., 2018). In another study, treating TBI model mice with the S1PR1 modulator CYM-5442 significantly reduced brain edema and neurological deficits (Zhang et al., 2022). CYM-5442 also inhibited lymphocyte migration for a short period of time but did not induce a sustained lymphocyte decrease (Zhang et al., 2022). In contrast, another study showed that treatment with fingolimod (1 mg/kg) significantly reduced the number of circulating lymphocytes and attenuated immune cell invasion of the damaged brain parenchyma, but failed to reduce the area of brain injury and improve neurological deficits in both the acute and chronic phases of brain injury in two different mouse models of TBI (Mencl et al., 2014).

## 6 Conclusion

**FIGURE 2:**
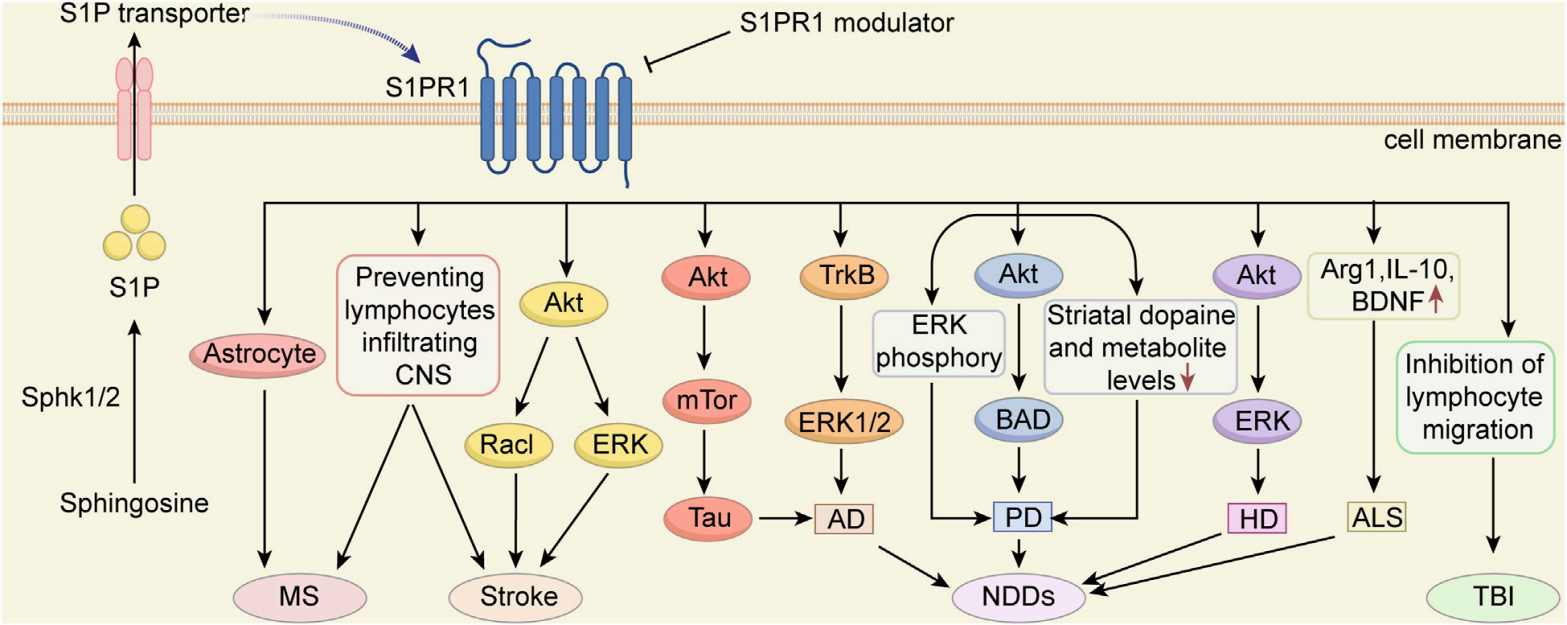
The potential mechanisms of S1PR1 modulators in protecting against CNS diseases S1PRs play a crucial role in normal CNS development, neural stem cell self-renewal, and differentiation. Among them, S1PR1 has emerged as a promising therapeutic target for various diseases. S1PR1 modulators exhibit significant treatment potential in conditions such as multiple sclerosis (MS), stroke, traumatic brain injury (TBI) and neurodegenerative diseases (NDDs), including Parkinson’s disease (PD), Alzheimer’s disease (AD), Huntington’s disease (HD) and Amyotrophic lateral sclerosis (ALS). This figure illustrates the potential mechanisms by which S1PR1 modulators exert protective effects on CNS diseases.

S1PR participates in cell survival, migration, phenotyping, activation, and proliferation in all biological systems and can modulate the pathophysiological processes of a variety of diseases. In this review, we summarized the effect of S1PR1 modulators on brain injury and provided an overview of the use of S1PR1 modulators for treating MS, stroke, NDDs, and TBI (Figure 2). As more S1PR subtype modulators are developed, their role in the treatment of brain injury should be investigated. In addition, we anticipate that more S1PR modulators with clinical potential will be useful for treating various diseases.

## Abbreviations

S1P, sphingosine 1-phosphate; CNS, central nervous system; MS, multiple sclerosis; BBB, blood-brain barrier; AD, alzheimer’s disease; GMH, germinal matrix hemorrhage; MACO, middle cerebral artery embolization; NDDs, neurodegenerative diseases; PD, parkinson’s disease; HD, huntington’s disease; ALS, amyotrophic lateral sclerosis; TBI, traumatic brain injury.
